# Supplementary material for: Deletion of wfs1 Impairs Oligodendrocyte Precursor Cells Dorsal Distribution and Myelination Through the wfs1-hmgcs1 Axis in Zebrafish
Source: Biology (Basel). 2026 Aug 16;15(16):1402. doi: 10.3390/biology15161402 (PMC13510151; doi:10.3390/biology15161402)
Supplement: Supplementary file 1 [file biology-15-01402-s001.zip › File S1-Transcriptome sequencing data and differentially expressed genes (DEGs) in wfs1-- zebrafish at 5 dpf/1.GO/wfs1KOvsWT/DOWN/wfs1KOvsWT.down_GObar_mf.pdf]

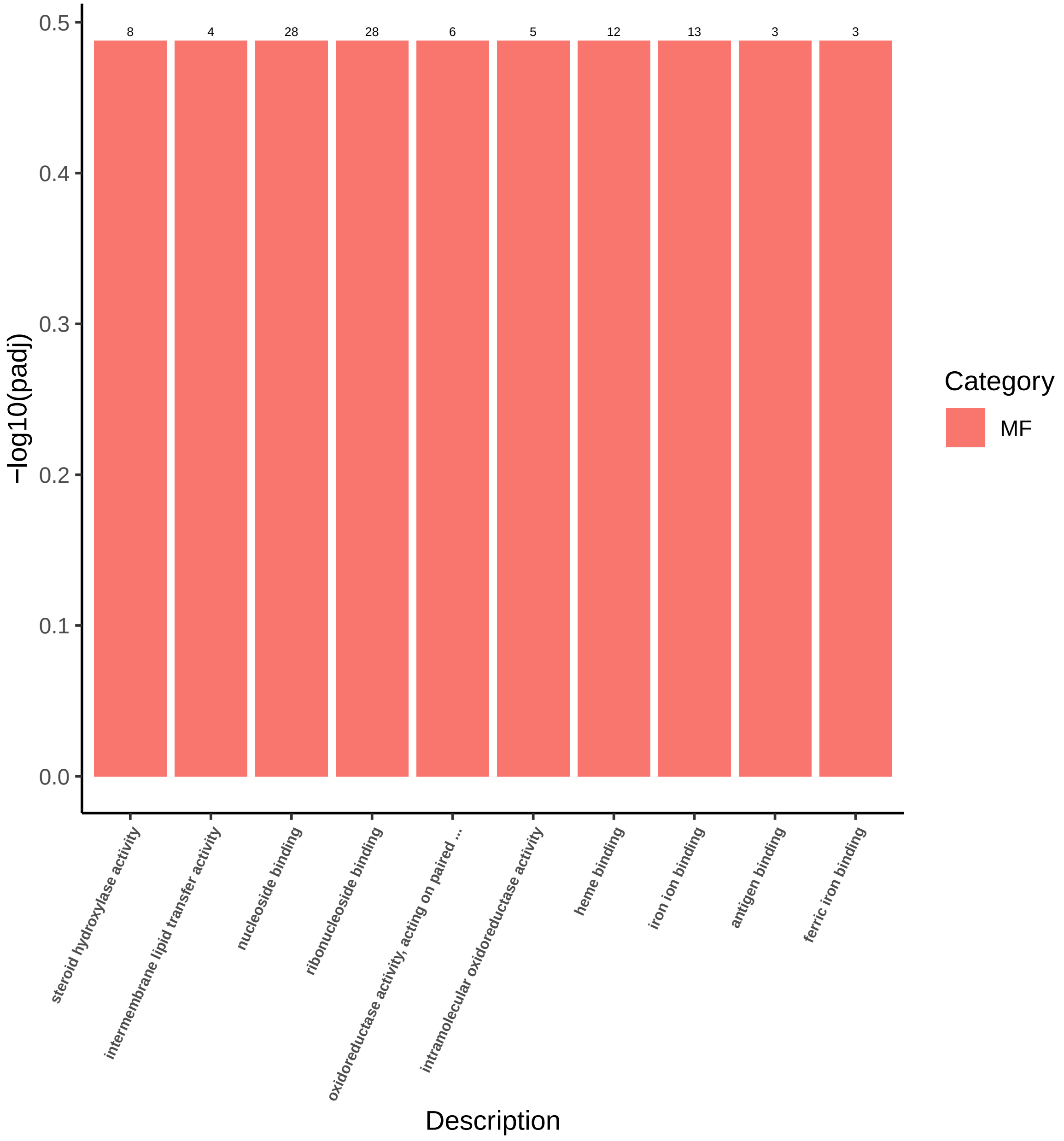

−log10(padj)
0.5
0.4
0.3
0.2
0.1
0.0
8
4
28
28
6
5
12
13
3
3
steroid hydroxylase activity
intermembrane lipid transfer activity
nucleoside binding
ribonucleoside binding
oxidoreductase activity, acting on paired ...
intramolecular oxidoreductase activity
heme binding
iron ion binding
antigen binding
ferric iron binding
Description
Category
MF
